# Greenhouse gas emissions resulting from conversion of peat swamp forest to oil palm plantation

**DOI:** 10.1038/s41467-020-14298-w

**Published:** 2020-01-21

**Authors:** Hannah V. Cooper, Stephanie Evers, Paul Aplin, Neil Crout, Mohd Puat Bin Dahalan, Sofie Sjogersten

**Affiliations:** 1 School of Biosciences, University of Nottingham, College Road, Leicestershire, Loughborough, LE12 5RE UK; 2 School of Natural Sciences and Psychology, Liverpool John Moores University, Byrom Street, Merseyside, Liverpool, L3 3AF UK; 3 School of Environmental and Geographical Sciences, University of Nottingham Malaysia Campus, Jalan Broga, Semenyih, 43500 Selangor Darul Ehsan Malaysia; 4 Department of Geography and Geology, Edge Hill University, St Helens Road, Ormskirk, Lancashire, L39 4QP UK; 5 Selangor State Forestry Department, Jabatan Perhutanan Negeri Selangor, Tingkat 3, Bangunan SSAAS, 40000 Shah Alam, Selangor Malaysia

**Keywords:** Climate-change mitigation, Environmental impact, Agriculture

## Abstract

Conversion of tropical peat swamp forest to drainage-based agriculture alters greenhouse gas (GHG) production, but the magnitude of these changes remains highly uncertain. Current emissions factors for oil palm grown on drained peat do not account for temporal variation over the plantation cycle and only consider CO_2_ emissions. Here, we present direct measurements of GHGs emitted during the conversion from peat swamp forest to oil palm plantation, accounting for CH_4_ and N_2_O as well as CO_2_. Our results demonstrate that emissions factors for converted peat swamp forest is in the range 70–117 t CO_2_ eq ha^−1^ yr^−1^ (95% confidence interval, CI), with CO_2_ and N_2_O responsible for ca. 60 and ca. 40% of this value, respectively. These GHG emissions suggest that conversion of Southeast Asian peat swamp forest is contributing between 16.6 and 27.9% (95% CI) of combined total national GHG emissions from Malaysia and Indonesia or 0.44 and 0.74% (95% CI) of annual global emissions.

## Abstract (web summary)

The magnitude of greenhouse gas emissions from land use change on tropical peatlands is unclear. Here, the authors measure greenhouse gas fluxes throughout the conversion from peat swamp forest to oil palm plantation, and estimate the contribution to regional and global emissions.

## Introduction

**Fig. 1:**
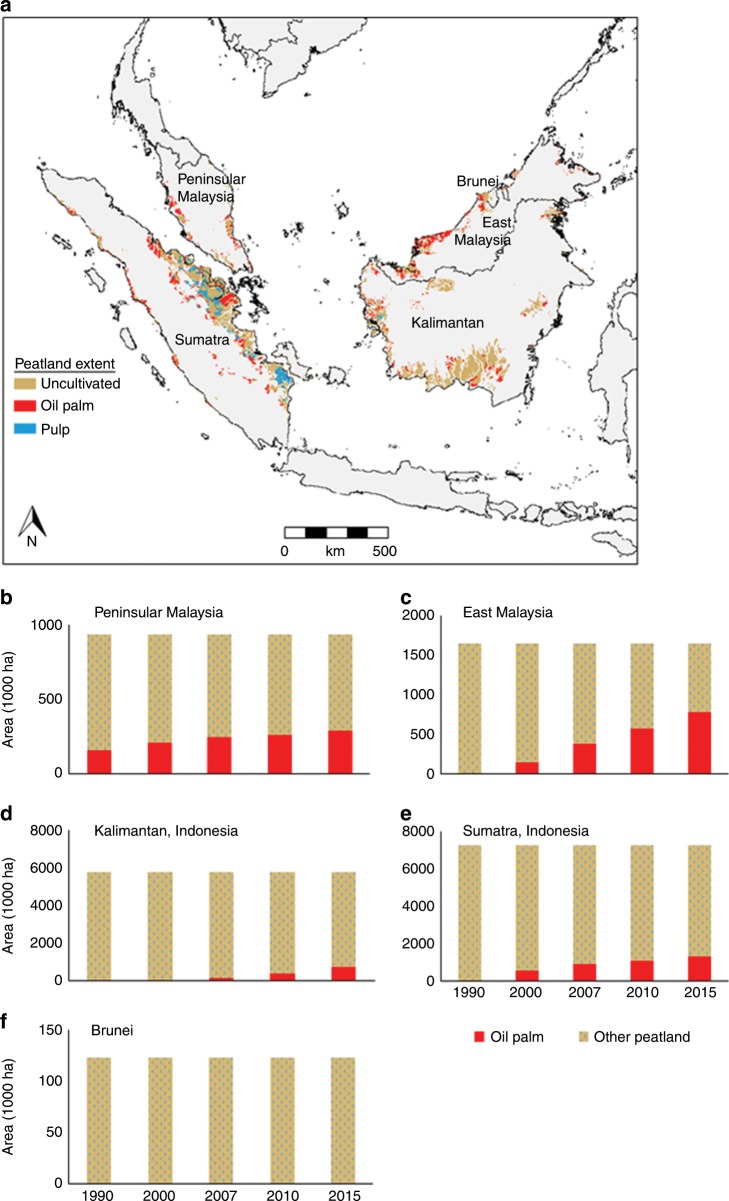
Peatland land use and land use change. **a** Map of peatland use showing the three main land use classes found in peatland areas in the region: uncultivated, oil palm and pulp plantations; and changes in area of oil palm plantation on peatland in **b** Peninsular Malaysia, **c** East Malaysia (Sarawak and Sabah), **d** Kalimantan, Indonesia, **e** Sumatra, Indonesia and **f** Brunei from 1990 to 2015. Note, pulp wood plantation, quite common in Sumatra though not elsewhere in the region, is incorporated into the ‘other peatland’ category in graphs **b** to **f**. Data source: Miettinen et al.^4^ and J. Miettinen pers. comm. Source data are provided as a Source Data file.

Tropical peat swamp forests hold ca. 20% (105 Gt) of global peatland Carbon (C)^1–3^. However, the contribution of peat swamp forests to C storage is currently under threat from large-scale expansion of drainage-based agriculture including oil palm and pulp wood production on peatlands (Fig. 1)^4^. Draining peatlands increases the oxygen levels in the soil, which in turn increases the rate of decomposition of organic material, resulting in high CO_2_ emissions from drained peatlands^5–7^. In addition to CO_2,_ peatlands also emit the powerful greenhouse gases (GHG) CH_4_ and N_2_O^8–10^. The emissions of these gases are known to be strongly influenced by land use change and drainage^9,11–13^. However, the magnitude of these effects on emissions is currently poorly quantified^14^, even though tropical agricultural peatlands have been highlighted as global hotspots for N_2_O emissions with implications for nitrogen management^14^. One of the most contentious issues in quantifying the environmental impacts of peat swamp forest conversion has surrounded the period during and immediately after conversion. High CO_2_ emissions in the first five years after conversion of peat swamps to agriculture were first estimated by Hooijer et al.^15^ on the basis of subsidence measurements. However, there have been no corresponding direct measurements of GHG emissions with which to compare these findings. The lack of data is partly due to the challenge of accessing sufficiently large areas of plantation at different stages of conversion to enable robust evaluation of the impacts of conversion. Existing studies either focus on single sites or make comparisons between forests and mature oil palm plantations without accounting for the conversion process, or focus on only one or two of these GHGs,^11,12,16^ making it difficult to calculate overall emissions factors from oil palm plantations. However, rapid loss of labile carbon following conversion implies that in situ emissions during the early phase of conversion will be higher than from mature plantations^17^.

This uncertainty has global consequences; a recent EU report on the social and environmental impacts of palm oil concluded: “the total GHG emissions from palm oil-related land use change is unknown”^18^. This highlights that data on GHGs from across the life cycle of oil palm plantations is urgently needed to underpin policy that supports the development of sustainable land management practices. The objective of this study is to calculate the global warming potential (GWP; i.e., expressed as CO_2_ eq.) resulting from conversion of peat swamp forest to oil palm plantation via direct measurement of GHG fluxes at a tropical peatland in North Selangor state, Malaysia.

The North Selangor peat swamp forest contains large areas of forest cover and high water tables^19^. However, it is encroached by oil palm plantations at various stages of development^19,20^. To determine the impact of conversion from peat swamp forest to oil palm plantation with regards to GHG emissions, we selected four stages of conversion that were present in the study areas: secondary forest, recently drained but uncleared forest, cleared and recently planted young oil palm plantation, and mature oil palm plantation (see Methods for a detailed site description). Previous work at the site shows that conversion strongly impacts soil physical properties, carbon storage and quality, with depletion of labile carbon acting as a strong control of both CO_2_ and CH_4_ production under anaerobic conditions^17,19^. Within each of these four conversion stages, we determine CO_2_, CH_4_ and N_2_O^21^ fluxes, as well as water table position, soil physio-chemical and vegetation properties, as they are important controls of GHG emissions. We show large emissions of CO_2_ and N_2_O following conversion while CH_4_ fluxes were reduced. CO_2_ emission fluxes are greatest during the drainage stage while N_2_O emissions were greatest in young oil palm plantations. The impact of conversion from forest to oil palm when combining CO_2_, CH_4_ and N_2_O fluxes increased the GWP, from 1435 tCO_2_ eq ha^−1^ at forest sites to 2744 tCO_2_ eq ha^−1^ over the 30-year life span of an oil palm plantation. This equates to an emission factor between 70–117 t CO_2_ eq ha^−1^ yr^−1^ (95% confidence interval, CI).

## Results and discussion

### Greenhouse gas emissions following land use conversion

**Fig. 2:**
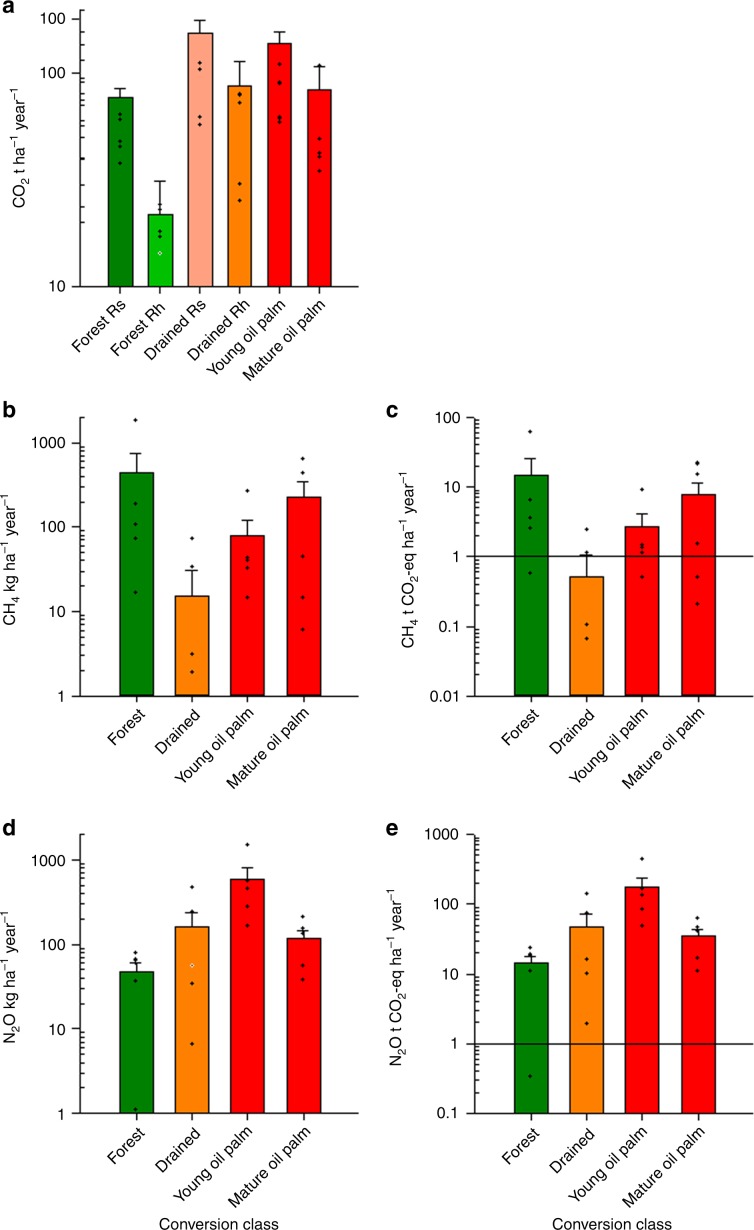
Greenhouse gas emissions across different land conversion stages. **a** CO_2_, **b** and **c** CH_4_, and **d** and **e** N_2_O fluxes from the four class conversion stages expresses as mass fluxes of each individual gas and as CO_2_ equivalents (i.e., after accounting for the global warming potential of each of the three gases). At forest and drained sites both heterotrophic respiration (Rh) and overall soil respiration (Rs); at the oil palm sites only Rh was measured. Mean and SEM are shown, *n* = 5. Note that a negative CH_4_ flux data point at the drained class is not shown as a dot point in panel **b** and **c** due to the log scale. Source data are provided as a Source Data file.

The greatest CO_2_ fluxes occurred during the drainage and young oil palm stages, before declining in the mature oil palm stage (*F*_3,41_ = 5.27, *P* < 0.05, *SED* = 12.22 t ha^−1^ yr^−1^, mixed model; Fig. 2a). Observed emissions from the mature plantations are comparable with heterotrophic fluxes previously reported from mature oil palm plantations of a similar age to those investigated in our study^6,7^. Emissions were ca. 50% greater from young oil palm than mature oil palm, supporting the notion of a peak in heterotrophic CO_2_ fluxes^15,22^ during the initial phases of peat swamp forest conversion to oil palm plantation. The high CO_2_ fluxes during the drainage stage are probably due to a combination of root respiration^23,24^ from trees and high heterotrophic respiration in response to increased oxygen availability after drainage^24,25^. This increase in emissions was evident even when water tables were <40 cm^26^ below the ground surface as recommended by the roundtable for sustainable oil palm (RSPO) guidelines^27^. This suggests that current guidance regarding water tables is not sufficient to mitigate high CO_2_ fluxes.

CH_4_ fluxes were high at the forest site; the median was 76 kg CH_4_ ha^−1^ yr^−1^, above the regional average of 38–56 kg CH_4_ ha^−1^ yr^−1^ ^8,13^ and declined sharply with drainage and conversion to either close to zero or negative fluxes representing a weak sink of CH_4_ (*F*_3,97_ = 3.82, *P* < 0.05, *SED* = 11.59 tCO_2_ eq ha^−1^ yr^−1^, mixed model; Fig. 2b, c). Our data clearly show reduced CH_4_ fluxes following conversion, in line with findings across SE Asia where drainage-based agriculture reduce emissions by approximately one third^13^. Importantly, although there was a sharp drop in CH_4_ emissions initially, these subsequently increased in mature plantations, possibly as a result of increased waterlogging at sites due to subsidence and compaction of the peat^15,19,28^.

Emissions of N_2_O were lowest at the forest sites; the median was 41 kg N_2_O ha^−1^ yr^−1^. The fluxes are in the range of emissions reported from drained forests in Central Kalimantan (31 kg N_2_O ha^−1^ yr^−1^)^16^. However, N_2_O emissions during the conversion phase (drainage and initial plantation) were much higher; with medians of 54 kg N_2_O ha^−1^ yr^−1^, and 291 kg N_2_O ha^−1^ yr^−1^, for drained forest and young oil palm plantations, respectively (*F*_3,134_ = 17.2, *P* < 0.01, *SED* = 53.87 tCO_2_ eq ha^−1^ yr^−1^, mixed model; Fig. 2c, Table 2). Increased N_2_O emissions in agricultural areas are commonly observed in studies on rangelands, sago, mixed agriculture and oil palm on drained peat^12,16,29^. Importantly, maximum emissions occurred in young oil palm plantations where fluxes were several orders of magnitude higher than N_2_O fluxes reported from both mature oil palm and peatlands used for other types of agriculture e.g. acacia plantations (3.4 and 2.6 kg N_2_O ha^−1^ yr^−1^, respectively)^12,16^. The likely explanation for these high fluxes is a combination of (i) application of inorganic N fertilisers and planting of leguminous cover crops in plantations to increase soil N levels, (ii) large supplies of labile C, a requirement of denitrifier heterotrophs^8,12,16^, from the decaying organic material derived from the recently cleared forest, and (iii) weakly reducing redox conditions induced by lowered water tables^9^ (Table 1).

**Table 1:** Vegetation and soil properties under different land uses in North Selangor, Malaysia. Mean and standard error of the mean (in brackets) are shown.

Variable	Forest	Drained	Young oil palm	Mature oil palm
Basal area (m^2^ ha^−1^)	21.58 (3.99)	14.47 (2.08)	nd	nd
Tree density (stems ha^−1^)	286.66 (22.05)	281.46 (40.16)	143 (n/a)	143 (n/a)
Soil moisture (%)	82.3 (28.0)	46.3 (22.0)	33.3 (9.0)	56.6 (21.0)
Soil temp (°C)	26.1 (0.52)	26.9 (0.71)	29.8 (0.49)	27.8 (0.85)
Bulk density (g cm^−3^)	0.10 (0.008)	0.07 (0.006)	0.12 (0.010)	0.12 (0.018)
Organic matter (%)	95.1 (0.99)	92.5 (0.83)	91.1 (1.69)	81.3 (3.91)
pH	3.6 (0.06)	3.7 (0.06)	3.7 (0.08)	3.9 (0.08)
Water table (cm)	6 (22)	−14 (23)	−39 (15)	−21 (18)
Peat depth (cm)	189 (130)	329 (43)	247 (43)	115 (112)

nd: no data

### Global warming potential following land use conversion

Over a 30-year period (i.e., the life span of an oil palm plantation accounting for the different stages of conversion), the annual CO_2_ loss from oil palm plantations was 53.0 t CO_2_ ha^−1^ yr^−1^ (39.2–73.0 t CO_2_ ha^−1^ yr^−1^ 95% CI; Table 2). This estimate is significantly lower than the 95 t CO_2_ ha^−1^ yr^−1^ annual average cited by the EPA^22^ and the International Council on Clean Transportation (ICCT)^30^, both of which are based largely on Hooijer et al.^15^. However our CO_2_ emission result is in line with the 60 t CO_2_ ha^−1^ yr^−1^ prescribed by the RSPO^26^ for sites with water tables of −40 to −60 cm (as derived from^30–32^).

**Fig. 3:**
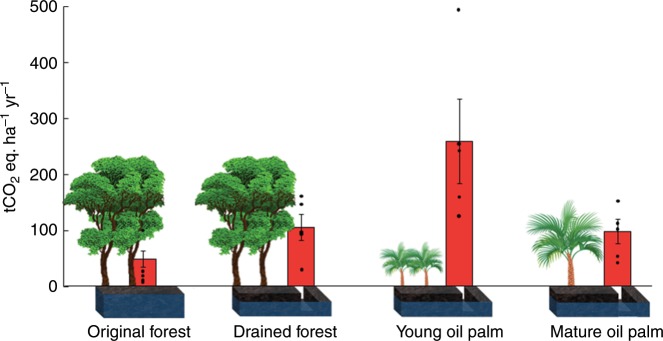
Emissions factors for each of the four land conversion stages expressed as tCO_2_ eq ha^−1^ yr^−1^ accounting for emissions of CO_2_, CH_4_ and N_2_O (i.e., accounting for the Global Warming Potential of each of these three gases). Mean and ±SEM are shown, *SED* = 62.99, *n* = 5. Source data are provided as a Source Data file.

Combining all emissions factors (i.e., for CO_2_, CH_4_ and N_2_O), GWP was greatest under the two early stages of conversion from peat swamp forest to oil palm plantation (Fig. 3), largely due to the high CO_2_ emissions from the drained sites and the high N_2_O emissions from the young oil palm sites (Fig. 2a, d and e). Significantly, our data demonstrate that it is misleading to compare GHG fluxes between only intact forest and mature oil palm without considering the contribution of the conversion process in the emissions factor.

To evaluate fully the net impact of forest conversion to oil palm agriculture on peatland GHG emissions, we compared the GWP, in CO_2_ eq, for forest and oil palm plantation over 30 years (Table 2). On this basis the conversion from forest to oil palm resulted in nearly doubling the GWP, from 1435 tCO_2_ eq ha^−1^ at forest sites to 2744 tCO_2_ eq ha^−1^ over the 30-year life span of an oil palm plantation. The annual emissions factor for oil palm plantations, accounting for all the GHGs and the different rates of emissions over the different phases of conversion, was 90 tCO_2_ eq ha^−1^ yr^−1^ (70–117 tCO_2_ eq ha^−1^ yr^−1^ 95% CI). Current emissions factors for oil palm grown on drained peat from the Intergovernmental Panel for Climate Change (IPCC) and US Environment Protection Agency are 40 and 95 tCO_2_ eq ha^−1^ yr^−1^, respectively, but those estimates only consider CO_2_.

**Table 2:** Emissions factors (EF) expressed as CO_2_ equivalents for the three GHG gases and the calculated combined global warming potential (GWP) expressed both per year (annual EF) and over the 30 year plantation production cycle (total EF).

	CO_2_ not adjusted to Rh t ha^−1^ yr^−1^	CO_2_ adjusted to Rh t ha^−1^ yr^−1^	CH_4_ tCO_2_ eq ha^−1^ yr^−1^	N_2_O tCO_2_ eq ha^−1^ yr^−1^	GWP tCO_2_ eq ha^−1^ yr^−1^
Forest	50.90 (4.82)^a^	18.73 (6.12)	14.78 (6.65)	13.51 (6.34)	47.83 (12.61)
Drained	88.49 (12.04)^a^	56.32 (13.15)	0.52 (0.23)	55.62 (21.47)	104.86 (22.88)
Young oil palm	81.37 (9.54)	81.37 (9.54)	2.68 (1.20)	182.42 (78.03)	258.53 (75.43)
Mature oil palm	54.41 (13.25)	54.41 (13.25)	7.67 (3.43)	34.34 (15.78)	97.36 (21.68)
SED	13.15	12.22	11.59	53.87	62.99
Annual EF^b^	tCO_2_ eq ha^−1^ yr^−1^	53.1 (39.2–71.0)			90 (69.9–117.5)
Total EF^b^	tCO_2_ eq ha^−1^	1591 (1177–2130)			2743.8 (2100–3524)

Mean, standard error of the mean (in brackets) and SEDs are reported for the different conversion stages. Mean and 95% CI (in brackets) are reported for the annual and total emissions factors.

^a^Soil emissions represent total emissions from both heterotrophic and autotrophic sources

^b^Based on the Monte Carlo simulations

Our data show it is important to include CH_4_ and N_2_O fluxes into calculations of emission factors as excluding these results in a serious underestimation at some conversion stages. Higher CH_4_ and N_2_O emissions following conversion of subtropical peatlands to agricultural land has been shown to increase the GWP substantially^28,29,33^. However, at 5.3 tCO_2_ eq ha^−1^ yr^−1^, the GWP reported from temperate peatland pasture systems was considerably lower compared to the rates we show for conversion to oil palm plantation^29^. The estimates provided here represent an important improvement over previous emission factors. In particular, these findings account for CH_4_ and N_2_O and include an uncertainty estimate and account for the process of conversion. The uncertainty associated with these estimates should be noted and the impact of temporal (e.g., diurnal cycles in fluxes, seasonality, plantation age) and spatial (e.g., site management and pedology) variability need to be better understood if this uncertainty is to be further reduced.

### Regional greenhouse gas emissions estimates

Using our emission factor estimation, annual GHG emissions from conversion of peat swamp forest to oil palm plantations in SE Asia are calculated to be 0.39 Gt CO_2_ eq yr^−1^ (based on the area of peat swamp forest currently converted to oil palm plantation, 3.1 × 10^6^ ha,^4^ and the emission factor of 90 t CO_2_eq ha^−1^ y^−1^ reported above). As the IPCC annual global estimate for GHG emissions in 2010 was 49 GtCO_2_ eq yr^−1^(ref. ^34^), we estimate that conversion of peat swamp forest contributes ca. 0.57% (0.44–0.74%, 95% CI) of the global total GHG emissions each year. At the regional scale, with combined annual GHG emissions from Indonesia and Malaysia of 2.66 GtCO_2_ eq yr^−1^^35^, the emissions from conversion to oil palm makes a substantial contribution at ca. 21.3% (16.6–27.9%, 95% CI) to the total regional GHG emissions. Note that this calculation does not account for the varying ages of oil palm plantation in the area and is based on the assumption that all oil palm plantation is established by conversion from forest. However, based on an average 25–30 year cropping cycle for oil palm, and existing land cover data, it is likely the majority of peatland oil palm plantations are first generation^4^.

Currently GHG emissions from tropical peatlands that have been drained and converted for plantation agriculture (e.g. oil palm) are not considered in GHG emissions budgets by the UN Framework Convention on Climate Change (UNFCCC)^36^. However, the overall sustainability of biofuel derived from palm oil, especially for companies actively engaged in peatland conversion, is still under consideration by bodies such as the EU Transport Agency^30^ and the US Environmental Protection Agency^37,38^. Similarly, a vote by members of the European Parliament in April 2018 prohibited sales of biofuels made from vegetable oils by 2020 in order to meet its 2020 climate goals. The grounds for the ban was that palm oil derived biofuels do not align with EU directives which state that biofuels should not contribute to global deforestation^37^. These policy initiatives clearly illustrate the requirement for appropriate emission factors.

In conclusion, we have demonstrated that the climate impact of converting tropical peatland to oil palm plantation is greatest during the early stages of conversion. This shows that simple comparison between forest and mature oil palm plantations do not appropriately account for the emissions throughout the oil palm plantation cycle. In the current study, when emissions of the three main GHGs are accounted for over a 30-year plantation cycle, the emission factor was nearly doubled (expressed in CO_2_ eq yr^−1^) compared to the emissions factor based solely on CO_2_ fluxes (Table 2) although considerable uncertainties remain due to temporal and spatial variability. Continued deforestation and conversion of peat swamp forest to oil palm plantation will result in release of globally significant amounts of GHGs to the atmosphere over the next decades.

## Methods

### Study area

This study was carried out in North Selangor Peat Swamp Forest (NSPSF), Malaysia, an ombrotrophic peatlands, which contains large areas of forest cover and high water tables^19^. The area of NSPSF is 73,600 ha, the site is split into two separate management areas: The northern part form the 50,100 ha Sungai Karang Forest Reserve while the southern parts is in the 23,500 ha Raja Musa Forest Reserve^39^. The area has a history as state land which resulted in logging and deforestation of part of the peatland. Logging was much reduced after 1990 when the area was designated as a reserve^40^. The logging history of the area means that the condition of the forest varies considerably. Furthermore, the forest is traversed by a network of ca 500 km of narrow canals that previously were used for transporting timber. Some areas of the reserve still has good quality dense forest as they have not been logged for *ca*. 40 years and these were selected as the forest sites used for this study^41^. The forest is largely comprised by the following tree species: *Macaranga pruinosa*, *Campnospermacoriaceum*, *Blumeodendron tokbrai*, *Shorea platycarpa*, *Parartocarpus venenosus*, *Ixora grandiflora*, *Pternandra galeata*, *Crytostachys sp*., and *Pandanus atrocarpus*^42^.

The site first became forested during the early Holocene when it was colonised by mangroves, these were over time replaced by fresh water vegetation and forest communities. At the base of the peatland are grey marine clays over which peat deposits has accumulated to up to 5 m depth^42^. The mean annual rainfall is more than 2000 mm per year, with a dry period in June, when rainfall is between 76 to 191 mm, the largest amount of rain falls in November when precipitation is 185 to 414 mm^43^. The mean annual air temperature is 28.5 °C and the humidity is 77.2%^44^. Although the majority of the reserve remains forested, it is encroached upon at the periphery by oil palm plantations at both early and mature stages^45^. As part of the study we chose four land use types that represent the stages of conversion from peat swamp forest to oil palm plantation, namely: (1) secondary ‘forest’ – these sites were located in areas with low recent anthropogenic impact, though the whole forest reserve was selectively logged during the 20^th^ century; (2) recently ‘drained’ but not cleared forest—at these sites, drainage took place ca. 6 months prior to sampling, drainage ditches were ca. 2 metres deep and 200–300 m apart; (3) drained, cleared and recently planted ‘young oil palm’ plantation which was established ca. six months prior to sampling—at the time of sampling the oil palms were 0.5 to 1 m tall; and (4) ‘mature oil palm’ first generation plantations, which were 10–15 years old with most trees between 8 and 12 m in height (see Tonks et al.^19^ and Table 1 for details).

### Field sampling and laboratory analysis

Within each of these four land use types, five sites were selected. At each site, a 30 by 30 m plot was established, the location of each plot was determined using random coordinates. Within the plot, three replicate static head space chambers of known volume (11.5 dm^3^) and area (425 cm^2^) were inserted to 2 cm depth and used to sample CO_2_, CH_4_ and N_2_O^21^ through a Suba seal; thus there were 60 sampling locations for each sampling event. At the young and mature oil palm plantations (stages 3 and 4), samples were collected 3.5 m away from the palm trunk to ensure negligible contribution of autotrophic respiration to measured surface CO_2_ fluxes^6,7,32^. During soil sampling and chamber installation, observations of ca. 10 cm diameter soil samples collected at 0–10 cm depth and ca. 5 cm diameter soil samples collected from 40–50 cm depth from each plot confirmed that there were no oil palm roots at the sampling locations. This was not possible at the forest sites; instead we applied a 63.2% contribution of autotrophic respiration to correct the surface fluxes at the secondary and drained forest sites^24,46^. Very similar rates of autotrophic respiration have been reported from peat swamp forest in SE Asia and the Neotropics^24,46^. Gas sampling was repeated three times at the forest, young oil palm and mature oil palm sites during the 2014 wet season (October-December); repeat sampling was not possible at the drained sites due to access problems. The overall sampling programme resulted in 150 independent sampling points across the 20 different sites. Samples were collected at 0, 2, 6 and 10 min using hypodermic needles and 20 ml syringes (25 G × 1”, TERMO, UK). The air within the chambers was gently mixed prior to sample extraction using the syringe and needle. Samples were then injected into pre-evacuated 12 ml glass vials (Exetainers, Labco, UK). All samples were shipped to the University of Nottingham, UK for gas chromatography analyses.

Vials were discarded for chromatographic analyses if overpressure was absent (<5 out of a total of 600 vials). CO_2_, CH_4_ and N_2_O concentrations were determined using a single injection system with a 1 mL sample loop that passed the gas sample using N_2_ as carrier through a non-polar methyl silicone capillary column (CBP1-W12-100, 0.53 mm I.D., 12 m, 5 mm; Shimadzu UK LTD, Milton Keynes, UK) and porous polymer packed column (HayeSep Q 80/100). Thermal conductivity (TCD), flame ionisation (FID) and electron capture (ECD) detectors were used to measure CO_2_, CH_4_ and N_2_O concentrations, respectively. Flux calculations were made using the ideal gas law and all samples were checked that gas accumulation in the head spaces were linear over time.

Drainage and lowered water tables are key features of conversion of peat swamp forest to oil palm plantation and can affect GHG production strongly^5^. Therefore, water table depth was measured at the time of GHG sampling at each field plot using dip wells. To explore longer term variation in water table depth, monthly variation was measured over a two-year period using dipwells at two locations in the secondary forest.

Structural woody vegetation measurements were taken at the field plots. At the forest and drained sites, diameter at breast height (DBH; calculated via circumference measured at a height of 140 cm) was calculated for all trees with DBH > 10 cm. From these data, basal area and stem density per ha were calculated. At young oil palm and mature oil palm sites, trunk height of all trees was estimated using a clinometer and tape measure.

Surface peat samples (10×10×10 cm volume) were collected using a bread knife at the first sampling event adjacent to the gas sampling locations in each of the field plots. Samples were placed in plastic bags and shipped to the University of Nottingham, UK. Prior to analysis, samples were cold stored at 4°C. Surface peat pH was determined by mixing 5 cm^3^ of field-wet peat in 12.5 cm^3^ of distilled water in centrifuge tubes and leaving on a rotary shaker overnight, before measuring with a pH 209 benchtop pH metre (Hanna Instruments Ltd.) and combination pH electrode.

Gravimetric water content was assessed by oven drying the peat samples at 105 °C for 48 h. The peat mass was recorded before and after oven drying and applied to Eq. (1). Bulk density was determined using the oven dried mass and known volumes, as in Eq. (2). Organic matter content was quantified using the loss on ignition method. 5 g samples of oven dried, ball milled peat were weighed into porcelain crucibles, before being placed in a Carbolite AAF muffle furnace (Carbolite Ltd.) at 550 °C for 4 h. The weight of ash left after ignition was recorded and Eq. (3) was used to determine the percentages of organic matter.

1 $$\theta = \frac{{M_{\mathrm{w}} - M_{\mathrm{d}}}}{{M_{\mathrm{d}}}} \times 100$$

Where *θ* is the gravimetric water content, dry weight basis (%); *M*_w_ is the mass of wet peat (g); and *M*_d_ is the mass of oven dry peat (g).

2 $$\rho _{{\mathrm{bulk}}} = \frac{{M_{\mathrm{d}}}}{V}$$

Where *ρ*_bulk_ is the bulk density, dry weight basis (g cm^−3^); *M*_d_ is the mass of oven dry peat (g); and *V* is the volume of the peat core (cm^3^).

3 $${\mathrm{OM}} = \frac{{M_1 - M_2}}{{M_1}} \times 100$$

Where OM is the organic matter content (%); *M*_1_ is the mass of oven-dry peat (g); and *M*_2_ is the mass of ash left after ignition (g).

### Data analysis and calculation of emissions factors

Mixed models using residual maximum likelihood method (REML) were used to test for differences in GHG fluxes between land uses. Land use type and time were used as fixed effects, ‘plot’ was fitted as a random effect. The spatial subsamples within each site were averaged at each time point before statistical analysis. GHG flux data were assessed for normality and subsequently transformed logarithmically. Statistical analysis was conducted using Genstat (version 15.1.0). We used the means from the measuring period (November – December 2014) to estimate annual fluxes, calculating GWP (CO_2_ equivalents) using equivalent values for CH_4_ and N_2_O of 34 and 298, respectively^47^. To determine whether variation in the water table affected the measured fluxes, linear relationships between gas flux and water table position for each combination of GHG (CO_2_, CH_4_, N_2_O) and land use (forest, drained, young oil palm, mature oil palm) were tested. There were no significant relationships between short term site water table fluctuations and any of the three GHGs in line with findings by Carlson et al.^48^, hence the mean measured gas fluxes were used to calculate emissions factors across the year. Note that although measurements were made during the wet season, water tables range widely between sites and over time including in the mature oil palm plantations which were flooded at some sampling time points (Table 1).

To estimate a confidence interval on emissions factors for the converted system we undertook a monte carlo analysis in which the emission rates for each GHG and conversion stage were sampled from the appropriate observed log-normal distribution. To quantify the GHGs emissions over the full oil palm cycle of 30 years the time dependent emission rate was linearly interpolated between the sampled rates for each conversion stage. For this, we assumed that emission rates changed from the secondary forest to the drained forest value over a six month period and then to the young oil palm rate over a further six months. The period over which emission changes from the young oil palm stage to the more stable mature oil palm was allowed to vary using a triangular distributed value which varied from 4 to 6 years after initial conversion with the maximum at 5 years. While these assumptions simplify the influence of plantation age on emissions, they are in line with the observed year of the actual (forest drainage and clearance) conversion process, the account by Hooijer et al.^15^ of subsidence stabilisation after ca. 5–6 years of drainage, and the subsequently stable subsidence rates at 4.2 mm yr^−1^ for the rest of the oil palm plantation life cycle reported for peatlands in Peninsular Malaysia^49^. The assumptions of comparable decomposition rates between ca. 5–6 and 30 years since conversion is supported by the paper by Dariah et al.^7^ who found no significant differences in CO_2_ emissions between 6 and 15 year old oil palm plantations, and the paper by Hooijer et al.^31^ which shows consistent subsidence rates after 6 years whereby subsidence indicates decomposition of peat. Indeed, Cooper et al.^17^ suggest rapid decomposition of labile carbon during the early stage of conversion followed by more gradual decomposition during the mature phase, supporting the notion of relatively consistent decomposition rates in the later stage of plantation when most of the labile carbon has already been metabolised by the microbial community and released to the atmosphere. Based on these assumptions, we calculated time integrated emissions factors (combining all three GHGs accounting for their contrasting GWP) for forest, drained, young oil palm and mature oil palm sites (Table 2).

### Reporting summary

Further information on research design is available in the Nature Research Reporting Summary linked to this article.

## Supplementary information

Description of Additional Supplementary Information

Supplementary Data 1

Reporting Summary

## Data Availability

All data are available on request from the authors. The source data underlying Figs. 1–3 are provided as a Source Data file; additional data are in Supplementary Data 1 file.

## Source data

Source Data
