# Author Correction: Greenhouse gas emissions resulting from conversion of peat swamp forest to oil palm plantation

**DOI:** 10.1038/s41467-020-15178-z

**Published:** 2020-04-01

**Authors:** Hannah V. Cooper, Stephanie Evers, Paul Aplin, Neil Crout, Mohd Puat Bin Dahalan, Sofie Sjogersten

**Affiliations:** 1 School of Biosciences, University of Nottingham, College Road, Leicestershire, Loughborough LE12 5RE UK; 2 School of Natural Sciences and Psychology, Liverpool John Moores University, Byrom Street, Merseyside, Liverpool, L3 3AF UK; 3 School of Environmental and Geographical Sciences, University of Nottingham Malaysia Campus, Jalan Broga, Semenyih, 43500 Selangor (Darul Ehsan) Malaysia; 4 Department of Geography and Geology, Edge Hill University, St Helens Road, Ormskirk, Lancashire, L39 4QP UK; 5 Selangor State Forestry Department, Jabatan Perhutanan Negeri Selangor, Tingkat 3, Bangunan SSAAS, 40000 Shah Alam, Selangor Malaysia

**Keywords:** Climate-change mitigation, Environmental impact, Agriculture

Correction to: *Nature Communications* 10.1038/s41467-020-14298-w, published online 21 January 2020.

The original version of this article contained an error in Fig. 2a, in which additional tick marks were added to the *y*-axis. This has been corrected in both the PDF and HTML versions of the article.

