# Supplementary material for: Greenhouse gas emissions resulting from conversion of peat swamp forest to oil palm plantation
Source: Nat Commun. 2020 Jan 21;11:407. doi: 10.1038/s41467-020-14298-w (PMC6972824; doi:10.1038/s41467-020-14298-w)
Supplement: Supplementary file 1 — Description of Additional Supplementary Information [file 41467_2020_14298_MOESM1_ESM.docx]

**Description of Additional Supplementary Files**

**File Name**: Supplementary Data 1
**Description:** Greenhouse gas flux data points, data on water table position, air temperature and percent ground surface covered by water.
