# Supplementary material for: Greenhouse gas emissions resulting from conversion of peat swamp forest to oil palm plantation
Source: Nat Commun. 2020 Jan 21;11:407. doi: 10.1038/s41467-020-14298-w (PMC6972824; doi:10.1038/s41467-020-14298-w)
Supplement: Supplementary file 3 — Reporting Summary [file 41467_2020_14298_MOESM3_ESM.pdf]

nature research

Corresponding author(s): Sofie Sjogersten
Last updated by author(s): Oct 1, 2019

Reporting Summary

Nature Research wishes to improve the reproducibility of the work that we publish. This form provides structure for consistency and transparency in reporting. For further information on Nature Research policies, see Authors & Referees and the Editorial Policy Checklist .

Statistics

For all statistical analyses, confirm that the following items are present in the figure legend, table legend, main text, or Methods section.

n/a	Confirmed	
☐	☒	The exact sample size (*n*) for each experimental group/condition, given as a discrete number and unit of measurement
☐	☒	A statement on whether measurements were taken from distinct samples or whether the same sample was measured repeatedly
☐	☒	The statistical test(s) used AND whether they are one- or two-sided<br>*Only common tests should be described solely by name; describe more complex techniques in the Methods section.*
☒	☐	A description of all covariates tested
☐	☒	A description of any assumptions or corrections, such as tests of normality and adjustment for multiple comparisons
☐	☒	A full description of the statistical parameters including central tendency (e.g. means) or other basic estimates (e.g. regression coefficient) AND variation (e.g. standard deviation) or associated estimates of uncertainty (e.g. confidence intervals)
☐	☒	For null hypothesis testing, the test statistic (e.g. *F*, *t*, *r*) with confidence intervals, effect sizes, degrees of freedom and *P* value noted<br>*Give P values as exact values whenever suitable.*
☒	☐	For Bayesian analysis, information on the choice of priors and Markov chain Monte Carlo settings
☒	☐	For hierarchical and complex designs, identification of the appropriate level for tests and full reporting of outcomes
☒	☐	Estimates of effect sizes (e.g. Cohen's *d*, Pearson's *r*), indicating how they were calculated

Our web collection on statistics for biologists contains articles on many of the points above.

Software and code

Policy information about availability of computer code

Data collection	N/A
Data analysis	Statistical analysis was conducted using Genstat (version 15.1.0).

For manuscripts utilizing custom algorithms or software that are central to the research but not yet described in published literature, software must be made available to editors/reviewers. We strongly encourage code deposition in a community repository (e.g. GitHub). See the Nature Research guidelines for submitting code & software for further information.

Data

Policy information about availability of data

All manuscripts must include a data availability statement. This statement should provide the following information, where applicable:

- Accession codes, unique identifiers, or web links for publicly available datasets
- A list of figures that have associated raw data
- A description of any restrictions on data availability

All data is available on request from the authors.

Field-specific reporting

Please select the one below that is the best fit for your research. If you are not sure, read the appropriate sections before making your selection.

☐ Life sciences ☐ Behavioural & social sciences ☒ Ecological, evolutionary & environmental sciences

For a reference copy of the document with all sections, see nature.com/documents/nr-reporting-summary-flat.pdf

Ecological, evolutionary & environmental sciences study design

All studies must disclose on these points even when the disclosure is negative.

Study description	Within each of these four land use types, five sites were selected. At each site, a 30 by 30 m plot was established, the location of each plot was determined using random coordinates. Within the plot, three replicate static head space chambers of known volume (11.5 dm3) and area (425 cm2) were inserted to 2 cm depth and used to sample CO2, CH4 and N2O21 through a Suba seal; thus there were 60 sampling locations for each sampling event.
Research sample	Air and soil samples
Sampling strategy	The spatial and temporal replication was based on the variability at a near by study site in Selangor and logistical contraints.
Data collection	Data was recorded in the field by the lead author. Gas analysis data from a laboratory instrument was checked indiviually for accurancy and downloaded. Data fiels were checked by Cooper.
Timing and spatial scale	Gas sampling was repeated three times at the forest, young oil palm and mature oil palm sites during the 2014 wet season (October-December); repeat sampling was not possible at the drained sites due to access problems. The overall sampling programme resulted in 150 independent sampling points across the 20 different sites. Samples were collected at 0, 2, 6 and 10 min using hypodermic needles and 20 ml syringes (25 G×1”, TERMO, UK). Three sub samples were collected from each plot at each sampling location.
Data exclusions	Data was checked if patterns in gas sample time series indicated leaks or ebullition. This was less than 2% of samples.
Reproducibility	The data is from a field study in an areas with variable climatic conditions. We repeated sampling over time three times.
Randomization	Sample locations were selected using random coordinates.
Blinding	Numerical codes were given samples for gas analysis. As we needed to check for the gas concentrations patterns over time we used set criteria to avoid bias.

Did the study involve field work? ☒ Yes ☐ No

Field work, collection and transport

Field conditions	Field work was carried out between Nov-Dec. Temperature was ca 30 degrees C and rainfall was intermittent. We report air and soil temperature as well as soil moisture and degree of surface flooding.
Location	This study was carried out in North Selangor Peat Swamp Forest (NSPSF), Malaysia, 3o36'24''N; 101o14'16''E, which contains large areas of forest cover and high water tables. This tropical ombrotrophic peat swamp covers 73,592 ha and comprises the 50,106 ha Sungai Karang Forest Reserve to the north and the 23,486 ha Raja Musa Forest Reserve to the south.
Access and import/export	The work in the NSPSF was carried out under permits granted to TROCARI lead by S Evers at the University of Nottingham, Malaysia Campus. All samples imported to the UK was under the University of Nottingham Defra licence.
Disturbance	Soil sample size was minimised (10*10*10) and the gas sampling head scapes was only gently inserted into the ground (to ca 2 cm depth).

Reporting for specific materials, systems and methods

We require information from authors about some types of materials, experimental systems and methods used in many studies. Here, indicate whether each material, system or method listed is relevant to your study. If you are not sure if a list item applies to your research, read the appropriate section before selecting a response.

Materials & experimental systems

n/a	Involved in the study	
☒	☐	Antibodies
☒	☐	Eukaryotic cell lines
☒	☐	Palaeontology
☒	☐	Animals and other organisms
☒	☐	Human research participants
☒	☐	Clinical data

Methods

n/a	Involved in the study	
☒	☐	ChIP-seq
☒	☐	Flow cytometry
☒	☐	MRI-based neuroimaging
